# An Innovative Approach for Extraction of Smoking Addiction Levels Using Physiological Parameters Based on Machine Learning: Proof of Concept

**DOI:** 10.3390/diagnostics15222839

**Published:** 2025-11-09

**Authors:** Muhammet Serdar Bascil, Irem Nur Iscanli

**Affiliations:** 1 Department of Biomedical Engineering, Faculty of Technology, Selcuk University, 42250 Konya, Turkey; 2 Department of Biomedical Engineering, Institute of Science, Selcuk University, 42250 Konya, Turkey; irmnnur@hotmail.com

**Keywords:** smoking addiction, physiological parameters, FTND, SMOTE, class-weighting, k-fold, PCA, machine learning

## Abstract

**Objectives**: Determining individuals’ addiction levels plays a crucial role in facilitating more effective smoking cessation. For this purpose, the Fagerstrom Test for Nicotine Dependence (FTND) is used all over the World as a traditional testing method. It can be subjective and may influence the evaluation results. This study’s key innovation is the use of physiological signals to provide an objective classification of addiction levels, addressing the limitations of the inherently subjective Fagerström Test for Nicotine Dependence (FTND). **Methods**: Physiological parameters were recorded from 123 voluntary participants (both male and female) aged between 18 and 60 for 120 s using the Masimo Rad-G pulse oximeter and the Hartman–Veroval blood pressure monitor. All participants were categorized into four addiction groups: healthy, lightly addicted, moderately addicted, or heavily addicted with the help of FTND. The recorded data were classified using Decision Tree, KNN, and SVM methods. SMOTE and class-weighting techniques were used to eliminate class imbalance. Also, the PCA technique was applied for dimensionality reduction, and the k-fold cross-validation method was employed to enhance the reliability of the machine learning algorithms. **Results**: Machine learning methods, when evaluated using the SMOTE with a (7380×7) sample of physiological signals recorded every 2 s from 123 participants, showed a high recall of 98.74%, specificity of 99.58%, precision of 98.79%, F-score of 98.74%, and accuracy of 98.75%. Also, it is extracted that there is a direct relationship between physiological parameters and smoking addiction levels. **Conclusions**: The study’s core novelty lies in leveraging non-invasive physiological signals to objectively classify addiction levels, addressing the subjectivity of the Fagerström Test for Nicotine Dependence (FTND). This study provides a proof-of-concept for the feasibility of using machine learning and physiological signals to assess addiction levels. The results indicate that the approach is promising.

## 1. Introduction

Tobacco addiction represents the most prevalent form of dependency worldwide. According to the World Health Organization (WHO), over 1.7 billion people, corresponding to approximately 22.3% of the global population, were reported as smokers in 2024, comprising 36.7% of men and 7.8% of women. Annually, more than 8 million people die from tobacco-related causes, including 1.3 million non-smokers and passive smokers [1,2]. This equates to an average of 19,100 tobacco-related deaths daily, underscoring its status as a critical global public health issue that demands urgent attention through early diagnosis and effective intervention strategies [1,2,3,4].

Traditionally, research in this field has heavily relied on self-report questionnaires like the Fagerström Test for Nicotine Dependence (FTND). Numerous studies have drawn conclusions based on FTND results. For instance, Marakoğlu et al. and Yazıcı and Ak used surveys and the Beck Depression Inventory to establish a significant link between smoking and higher depression scores among medical students [5,6]. Zincir et al. utilized FTND alongside other inventories to find that lower FTND scores were correlated with higher success rates in smoking cessation [7]. Çelepkolu et al. applied FTND to investigate demographic correlations, finding higher addiction rates in men and those who started smoking younger [8]. While practical, self-report tools are susceptible to bias, leading researchers to seek validation through objective biochemical markers like cotinine and exhaled carbon monoxide (CO) [9]. Although correlations between FTND scores and these biomarkers exist, they can be variable, highlighting a critical area for improvement in study design [10].

To overcome the limitations of subjective reporting, recent studies have turned to objective neuroimaging and electrophysiological methods. Notable works include Pariyadath et al. and Wetherill et al., who used SVM-based classification on resting-state fMRI data to identify brain regions associated with nicotine addiction with high accuracy [11,12]. Similarly, Eken et al. and Çay et al. achieved over 90% accuracy in classifying addiction levels using machine learning algorithms on ECG, PPG, and EEG data from participants assessed with FTND [13,14]. However, while powerful, technologies like fMRI and EEG are often high-cost, require controlled lab environments, and involve operator dependency, limiting their scalability for widespread screening or long-term monitoring.

In this context, the proliferation of wearable technology presents a significant opportunity for the objective and continuous monitoring of addictive behaviors. Wearables like smartwatches can provide passive physiological data that serve as digital biomarkers, offering valuable insights into substance use and craving symptoms. For example, studies have successfully used wearable sensor data to detect opioid use [15] and nicotine craving [16]. Similarly, other research demonstrates the analysis of cardiovascular and respiratory signals from wearables using machine learning [17,18].

Our study is founded on this promising paradigm. In contrast to complex and costly systems, the approach proposed here relies on low-cost, non-invasive physiological signals, such as SpO2, PR, RRp, Pi, and PVi, which can be easily recorded via wearable devices. This methodology not only offers scalability and accessibility but also holds the potential for continuous monitoring in real-world conditions outside the laboratory.

Given the limitations of subjective questionnaires, our study classifies participants’ dependency levels based on the FTND as a practical approach. However, a key future improvement will be to validate our model’s predictions against a biochemical “gold standard,” such as cotinine or CO measurements, as seen in recent studies [19,20]. This would significantly enhance the reliability of our class labels and the clinical validity of our model.

Therefore, this study should be regarded as a proof-of-concept investigation rather than a clinically validated diagnostic system. It aims not only to provide an alternative to subjective questionnaires but also to propose a portable, applicable, and reliable digital biomarker framework that overcomes the practical constraints of existing objective methods.

## 2. Materials and Methods

### 2.1. Dataset

**Figure 1:**
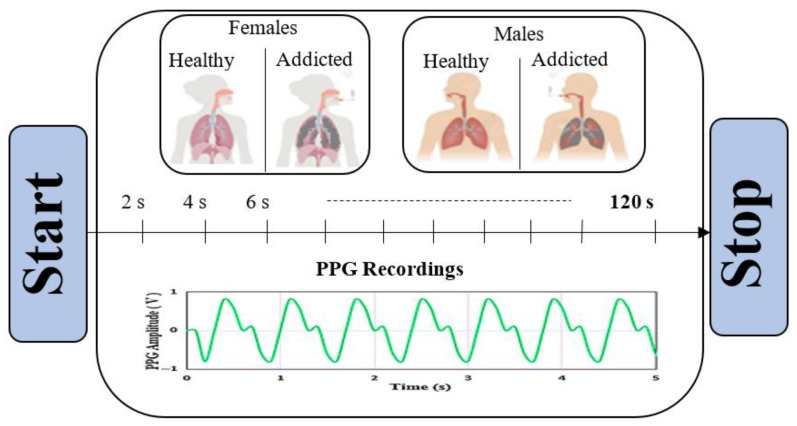
Data acquisition flow.

The dataset used in the study was collected from 123 volunteer participants (58 women, 65 men) aged 18–60, and no known health problems. Masimo Rad-G pulse oximeter [21] and Hartmann–Veroval [22] digital blood pressure devices were used for data recording. The corresponding dataset was recorded for 120 s for each participant and recorded every 2 s, including seven different physiological parameters: oxygen saturation (SpO2), pulse rate (PR), respiratory rate (RRp), perfusion index (Pi), pleth variability index (PVi), and blood pressure values (systolic-diastolic, mmHg). The data obtained for each person is every 2 s, as shown in Figure 1, and 60 different data points were obtained for each variable.

The PPG signals obtained from the participants were recorded at any time during the day for the non-smoking group, and the addicted group was recorded immediately after cigarette consumption (within 1–2 min of extinguishing the cigarette). This specific timing was chosen to capture the acute physiological response and regulation period following nicotine intake, which we hypothesized might correlate with dependency levels.

**Figure 2:**
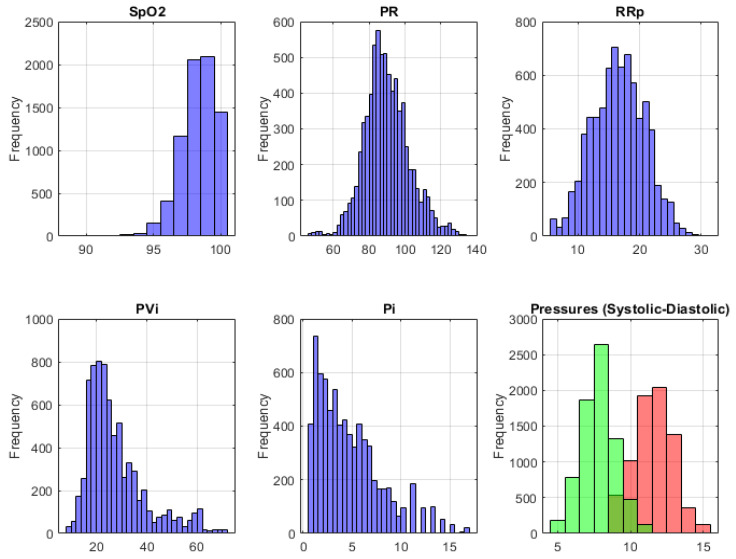
Frequency distribution of dataset on physiological parameters.

Further, 2400 different data points were recorded for the non-smoking group of 40 people (25 women, 15 men), and 4980 different data points were recorded for the addicted group (33 women, 50 men), with a total dataset of (7380×7). The systolic and diastolic blood pressure values obtained for each person were spread over the 120 s dataset belonging to the participant. Figure 2 shows the frequency distributions of the physical parameters.

As shown in Figure 2, the Spo2 data form a left-skewed histogram. This is an expected situation because the normal oxygen saturation value should be between 95 and 100%. The PR graph, which expresses the pulse value, exhibits an ordinary histogram. It is normal for this value to be between 60 and 100/min. Similarly, it can be said that the RRp graph, which expresses the respiratory rate, also exhibits an ordinary histogram. The average respiratory rate in a normal individual is between 10 and 20/min. PVi is known as the pleth variability index and is the parameter that allows the automatic monitoring of dynamic changes that occur during the respiratory cycle. It is normal for these parameter data to exhibit a left-skewed histogram because the normal values of the parameter should be between 0 and 100%. Similarly, the perfusion index Pi parameter, which expresses the circulation rate, also exhibits a left-skewed histogram. The range value of this parameter is known as 0.02–20% and its average value is 1.4%. In the last graph, it was normal for systolic (green) and diastolic (red) blood pressure change histograms to form an ordinary histogram because normal blood pressure values should be 80–120 mmHg. As a result, all parameters of forming the dataset showed frequencies within the expected limits.

**Figure 3:**
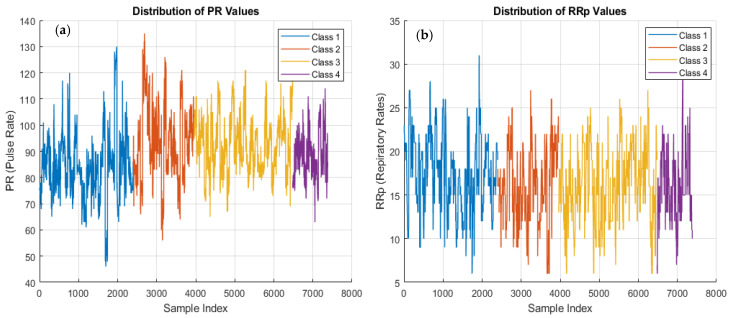
Distribution of pulse rates and respiratory rates: (**a**) PR, (**b**) RRp.

Upon examination of Figure 3, distinct dynamic behavior in pulse rate values is observed across all classes. The PR values generally fluctuate within a range of 60–120 beats per minute, which falls within clinically accepted normal limits. The variations observed between different classes can be considered potentially related to addiction levels.

The analysis of respiratory rate values shows that they generally range between 10 and 30 breaths per minute across all classes. These values largely align with the normal respiratory rate range for adults (12–20 breaths per minute). The prominent periodic fluctuations visible in the graph reflect the complex regulatory mechanisms of the respiratory system. The differences between classes may suggest information about pathophysiological mechanisms underlying variations in respiratory patterns.

Additionally, Table 1 provides a more detailed statistical analysis of the changes in basic vital parameters recorded from the participants.

An examination of Table 1 reveals a consistent decrease in oxygen saturation (SpO_2_) from Class 1 (98.1%) to Class 4 (87.1%). This trend indicates a decline in respiratory efficiency and suggests progressively unstable oxygenation with increasing dependency levels. In contrast, pulse rate (PR) demonstrates a corresponding increase (79.8 → 118.3 bpm). This pattern suggests a tendency for heart rate to rise with higher levels of addiction. Similarly, a progressive decrease is observed in PR SDNN (36.4 → 20.1 ms), a measure of short-term pulse rate variability. This finding indicates reduced heart rate stability associated with increased dependency. Furthermore, a marked increase is seen in both respiratory rate period (RRp) and its coefficient of variation (CV%) (16.8 → 27.9 s; 16.8% → 31.1%). This suggests that higher addiction levels are associated with more irregular and rapid breathing patterns. The overall pattern indicates a transition from efficient, stable respiration toward inefficient, labored breathing characterized by increased respiratory effort.

Table 2 displays the statistical variations in heart rate variability (HRV) analysis. The heart rate variability parameters show a gradual decline from Class 1 to Class 4. SDNN and RMSSD, which reflect overall and short-term HRV, respectively, demonstrate a steady decrease (SDNN: 35.8 → 19.3 ms; RMSSD: 30.2 → 13.1 ms). This pattern may indicate reduced autonomic adaptation and diminished parasympathetic activity in the higher dependency classes. The LF/HF ratio increased significantly (1.58 → 3.52), suggesting a shift in autonomic balance. Similarly, pNN50 and the HRV Index followed comparable declining trends, indicating a reduction in beat-to-beat variability. Overall, these results reveal a class-dependent decrease in HRV metrics, which can be interpreted as progressive autonomic imbalance and potential cardiovascular strain at higher levels of dependency.

Table 3 presents the analysis of respiratory dynamics, revealing a gradual shift toward rapid and unstable breathing patterns with increasing dependency levels. The respiratory rate demonstrates a marked increase from Class 1 to Class 4 (16.8 → 27.9 breaths/min). Furthermore, the respiratory rate coefficient of variation and breath interval standard deviation increased significantly (%16.8 → %31.1; 1.9 → 5.8 sec), indicating heightened respiratory irregularity. The Rapid Shallow Breathing Index (RSBI) shows a strong upward trend (37.3 → 85.8), potentially reflecting the onset of inefficient respiratory effort. Collectively, these results indicate that respiratory instability and a rapid, shallow breathing pattern are hallmark characteristics of higher dependency levels. When interpreted together, Table 1, Table 2 and Table 3 provide evidence that tobacco dependency is associated with a coordinated autonomic dysregulation, concurrently affecting both the cardiovascular and respiratory systems.

### 2.2. Method

**Figure 4:**
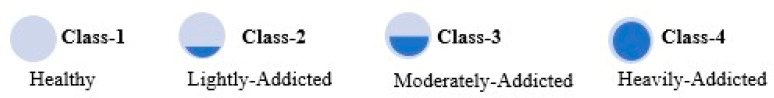
Evaluation and class scale.

**Figure 5:**
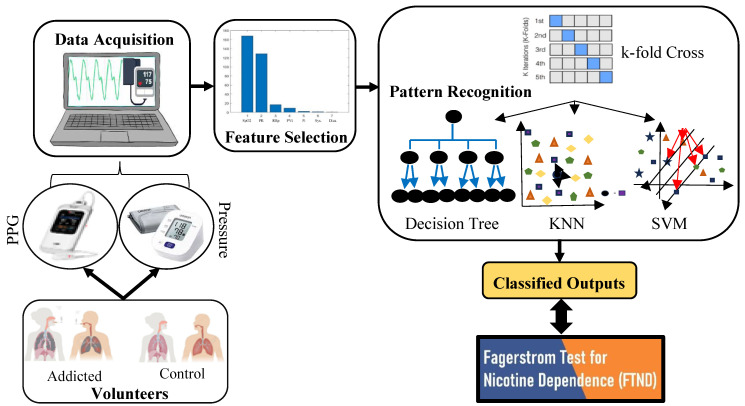
The methodology protocol of study.

Participants were categorized into four distinct groups based on their scores from the Fagerström Test for Nicotine Dependence (FTND). The FTND, originally developed by Karl-Olov Fagerström in 1978 [23] and updated in 1991 [24], is a widely used instrument. It is a subjective assessment that evaluates nicotine dependence based on smoking habits and behavioral patterns, rather than definitive medical criteria. The test comprises six questions, with item-level scores ranging from 0 to 3 or 0 to 1. The total score ranges from 0 to 10, where a higher score indicates a greater degree of dependence. In this study, each participant completed the FTND. Based on their total scores, participants were classified as follows (see Figure 4):

The labeled dataset was classified with the help of machine learning algorithms, as shown in the methodology protocol given in Figure 5, and the classified outputs were evaluated by comparing with the FNTD results.

### 2.3. Principal Component Analysis (PCA)

Principal Component Analysis (PCA) is a method applied to reduce the size of a large dataset with multiple variables. After applying the PCA method, the calculated variables are expressed as the principal components of the first variables of the dataset. In the PCA method, as the dataset gets closer to the normal distribution, PCA reaches the best performance value [25,26,27].

**Figure 6:**
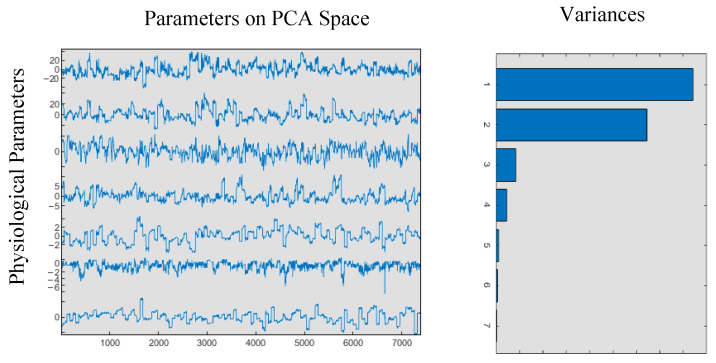
Physiological parameters on PCA space and their variances.

As presented in the histogram graph in Figure 6, the dataset consists of seven physiological parameters: SpO_2_, Pr, RRp, PVi, Pi, systolic, and diastolic pressure. PCA is employed to reduce the dimensionality of the features by analyzing the variances within the PCA space. It was determined that 5 principal components retain 99% of the total variance for all physiological parameters, as illustrated in Figure 5. Consequently, the original seven-dimensional dataset can be effectively represented using only five dimensions, preventing classifiers from processing redundant information. Based on the variance ratios, the dataset, originally sized at (7380×7), is reduced to (7380×5) within the PCA space while preserving 99% of the information.

### 2.4. Synthetic Minority Over-Sampling Technique (SMOTE)

The Synthetic Minority Over-sampling Technique (SMOTE) is a pioneering algorithm designed to mitigate the class imbalance problem in machine learning datasets. Unlike naive random oversampling, which simply duplicates minority class instances and can lead to overfitting, SMOTE generates synthetic examples to construct a more robust and generalized decision region for the minority class [28]. The core idea is to operate directly in the feature space, creating new, artificial instances that lie between existing minority class examples, thereby effectively enlarging the minority class cluster.

The algorithm’s mechanism is rooted in a controlled interpolation strategy between a seed instance and its neighbors. For a given minority class instance $x_{i}$, the process begins by identifying its k-nearest neighbors belonging to the same class, typically using the Euclidean distance metric. Subsequently, one of these neighbors, denoted as $\hat{x_{j}}$, is randomly selected. A synthetic data point $\tilde{x}$ is then generated along the line segment connecting $x_{i}$ and $\hat{x_{j}}$ in the feature space. This fundamental operation is mathematically defined by the following convex combination formula:

(1) $$\tilde{x}=x_{i}+\gamma \cdot (\hat{x_{j}}-x_{i})$$

Here, $\tilde{x}$ represents the feature vector of the new synthetic instance; $x_{i}$ is the feature vector of the i-th seed instance from the minority class; and $\hat{x_{j}}$ is the feature vector of the randomly chosen j-th nearest neighbor from the k neighbors of $x_{i}$ and γ is a random number drawn from a uniform distribution between 0 and 1. This scalar acts as an interpolation coefficient, determining the precise location of the new instance between $x_{i}$ and $\hat{x_{j}}$.

The vector difference ($\hat{x_{j}}-x_{i}$) defines the direction and magnitude of the line segment connecting the two original instances. Multiplying this vector by γ yields a random fraction of the segment. Finally, adding this scaled vector to the original point $x_{i}$ creates a new point that is a linear interpolation of the two. This procedure is applied iteratively and independently to each feature, generating a plausible synthetic instance within the convex hull defined by the minority class examples.

The primary advantage of this approach is that it forces the decision region for the minority class to become more general, as the classifier is exposed to a broader, more continuous distribution of minority samples rather than just the specific, and potentially sparse, original points. This helps in reducing the overfitting risk associated with mere replication and aids in constructing a more effective and generalized classifier [29]. The number of synthetic samples to be generated for each $x_{i}$ is controlled by an oversampling rate n, allowing for precise control over the final class distribution.

### 2.5. Class-Weighting Technique for Imbalanced Data

Class-weighting, also known as cost-sensitive learning, is a fundamental and widely adopted technique for addressing class imbalance. It operates at the algorithm level by assigning a higher misclassification cost to the minority class. This approach directly modifies the objective function of a learning algorithm, incentivizing the model to pay more attention to correctly classifying the minority class instances [30,31].

The core principle is to treat the classification errors unequally. Misclassifying a minority class instance is considered more detrimental than misclassifying a majority class instance. This is formally integrated into the model training process by assigning a weight to each class, which is typically inversely proportional to its class frequency.

The class weights are incorporated into the model’s loss function. For a general loss function L, the weighted loss L_weighted over a dataset of N samples is computed as follows:

(2) $$L_{weighted}=\frac{1}{N}\cdot \sum \left[w_{y_{i}}\cdot L\left(f(x_{i},y_{i})\right)\right]$$

where $L\left(f(x_{i},y_{i})\right)$ is the loss for the prediction $f_{x_{i}}$ compared to the true label $y_{i}$ for the i-th sample. $w_{y_{i}}$ is the weight assigned to the true class $y_{i}$ of the i-th sample. N is the total number of samples.

The most common strategy for determining the class weights $w_{j}$ for a class j is to use the inverse of the class frequency:

(3) $$w_{j}=\frac{N}{C\cdot N_{j}}$$

where N is the total number of training samples. C is the total number of classes. $N_{j}$ is the number of samples in class j.

This formula ensures that the total weight assigned to each class is balanced, preventing the model from being biased toward the majority class.

### 2.6. Machine Learning

Powerful methods commonly used in classification and pattern recognition tasks, such as Decision Trees, KNN, and SVM, have been implemented in this study. To improve the reliability of their accuracy assessments, the 5-fold cross-validation technique has been applied.

#### 2.6.1. k-Fold Cross-Validation Technique

**Figure 7:**
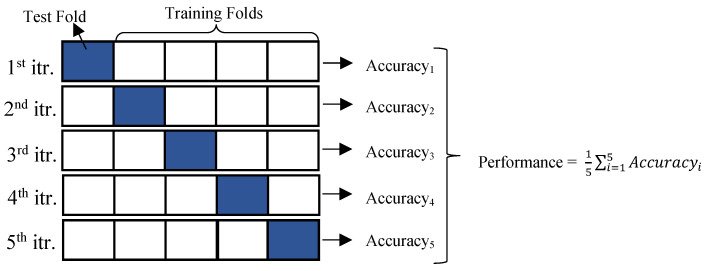
The process of 5-fold cross-validation.

In the k-fold cross-validation technique, the dataset is divided into multiple subsets (folds). In each iteration, one fold is designated as the test set, while the remaining folds are used for training the model. This process is repeated k times, ensuring that each subset serves as the test set exactly once, leading to a more robust and reliable evaluation of the model’s performance. Ultimately, the overall performance of the model is determined by averaging the evaluation metrics obtained from all iterations, as illustrated in Figure 7 [32]. The k-value was tested between 1 and 10, and the best classifier performances were obtained when k = 5.

The mathematical formulas for performance of k-fold are shown in Equations (4) and (5):

(4) $${Accuracy}_{i}=\frac{\sum_{i=1}^{\left|TF\right|}{TP}_{i}+{TN}_{i}}{{TP}_{i}+{TN}_{i}+{FP}_{i}+{FN}_{i}};$$

(5) $$Performance=\frac{1}{k}\sum_{i=1}^{k}{Accuracy}_{i}$$

here, TF represents the test fold used for classification, TP denotes true positive results, TN refers to true negative results, FP indicates false positive results, and FN corresponds to false negative results.

Additionally, performance evaluation metrics play a critical role in assessing the effectiveness and reliability of the classifiers [33]. These metrics are defined by Equations (6)–(9).

(6) $$Recall\;(Sensitivity)=\frac{TP}{TP+FN}$$

(7) $$Specificity=\frac{TN}{TN+FP}$$

(8) $$Precision=\frac{TP}{TP+FP}$$

(9) $$F\text{-}Score=2\times \frac{Precision\times Recall}{Precision+Recall}$$

Recall represents the proportion of true positive cases correctly identified by the model. Precision measures the accuracy of the model’s positive predictions. Specificity reflects the model’s effectiveness in correctly identifying negative classes. The F-Score, calculated as the harmonic mean of precision and recall, provides a balanced assessment of both the model’s accuracy and sensitivity.

#### 2.6.2. Decission Tree

The Decision Tree method introduced by Breiman is one of the supervised classification algorithms [34]. Root, node and leaves are the basic structures of a decision tree. It is based on the principle that each decision node in the tree is divided into two branches. Branching is continued until a new node is created [35].

The Gini Algorithm is used in the Decision Tree method. This algorithm provides classification by dividing the data into four categories: healthy, lightly addicted, moderately addicted, and heavily addicted. The relevant attribute values are grouped into four. The class values corresponding to the groupings obtained in this way are distributed. The Gini values for each attribute are calculated as in the Formulas (10)–(13);

(10) $${Gini}_{n-s}=1-{\sum^{k}}_{i=1}{\left(\frac{L}{\left|T_{n-s}\right|}\right)}^{2}$$

(11) $${{Gini}_{l-a}=1-\sum^{k}}_{i=1}{\left(\frac{K}{\left|T_{l-a}\right|}\right)}^{2}$$

(12) $${{{Gini}_{m-a}=1-\sum }^{k}}_{i=1}{\left(\frac{R}{\left|T_{m-a}\right|}\right)}^{2}$$

(13) $${Gini}_{h-a}=1-{\sum^{k}}_{i=1}{\left(\frac{P}{\left|T_{h-a}\right|}\right)}^{2}$$

here, k is the total number of classes in the data, T is the number of samples in a node, $\left|T_{n-s}\right|$ is the number of samples taken from the control group, $\left|T_{l-a}\right|$ is the number of samples taken from lightly addicted participants, $\left|T_{m-a}\right|$ is the number of samples taken from moderately addicted participants, $\left|T_{h-a}\right|$ is the number of samples taken from heavily addicted participants, L is the number of samples in the control group, K is the number of samples in the lightly addicted group, R is the number of samples in the moderately addicted group, and P is the number of samples in the heavily addicted group.

The classification formula is calculated as in Equation (14), where the relevant j value is the number of rows in the training set and n is the number of rows in the training set:

(14) $${Gini}_{j}=1/n(\left|T_{n-s}\right|\cdot {Gini}_{n-s}+\left|T_{l-a}\right|\cdot {Gini}_{l-a}+\left|T_{m-a}\right|\cdot {Gini}_{m-a}+\left|T_{h-a}\right|\cdot {Gini}_{h-a})$$

The smallest $\left|{Gini}_{j}\right|$ value calculated for the relevant attribute j is selected, thus the division is performed on this attribute. Finally, the process is continued by returning to the first step [36].

#### 2.6.3. K-Nearest Neighbor (KNN)

The K-Nearest Neighbors (KNN) algorithm is widely utilized in machine learning due to its simplicity and effectiveness. It classifies a new data point by analyzing the class labels of its nearest neighbors, thereby performing the classification task [37,38]. The fundamental principle of KNN involves identifying the K closest neighbors of a given data point and determining its class based on the majority class of these neighbors. In this process, the distances between data points are commonly measured using the Euclidean distance, as defined in Equation (15) [39].

(15) $$d\left(x_{i},x_{j}\right)=\sqrt{\sum_{k=1}^{n}{\left(x_{ik}-x_{jk}\right)}^{2}}$$

here, x_i_ and x_j_ represent the two data points being compared, x_ik_ and x_jk_ denote the k-th feature values of the respective points, and n is the number of features.

The number of neighbors (k was systematically varied across the set k = {1, 3, 5, 7, 9, 11, 15, 21} and the optimal value (k = 3) was determined using the elbow method, which balances model bias and variance [39]. To assess the impact of distance computation, Euclidean, Manhattan, Minkowski, and Cosine distance metrics were compared. Euclidean distance produced the best classification results [40].

#### 2.6.4. Support Vector Machine (SVM)

Support Vector Machine (SVM) is fundamentally based on the principle of identifying an optimal hyperplane that effectively separates the data [41]. The primary objective is to maximize the margin between different classes by constructing a hyperplane with the widest possible separation. The margin represents the distance between the hyperplane and the nearest data points, known as support vectors, which are crucial in determining the hyperplane’s position [41,42]. The SVM classification approach is mathematically formulated in Equations (16)–(19).

The training data (xi) belonging to two separated classes (yi),

(16) $$\left\{x_{i},y_{i}\right\},i=1,2,\ldots ,N,y_{i}\in \left\{-1,+1\right\},x_{i}\in R^{n};$$

represented with the optimal hyperplane,

(17) $$\left(w\cdot x_{i}\right)+b=0;$$

An optimal hyperplane with the largest margin can be formulated as follows:

(18) $$\begin{array}{c} x_{i}\cdot w+b\geq +1\;for\;y_{i}=+1\\ x_{i}\cdot w+b\leq +1\;for\;y_{i}=-1 \end{array}$$

which is equivalent to

(19) $$\left\{\begin{array}{c} w^{T}\varphi \left(x_{i}\right)+b\geq +1,\;if\;y_{i}=+1\\ w^{T}\varphi \left(x_{i}\right)+b\leq -1,\;if\;y_{i}=-1 \end{array}\to y_{i}\left[w^{T}\varphi \left(x_{i}\right)+b\right]\geq 1\right.$$

where φ(:) is a function which maps the input space into a higher-dimensional space.

The hyperparameter space tested in the grid search was constructed as follows: [‘linear’, ‘RBF (radial basis function)’, ‘polynomial’, ‘gaussian’] values for the kernel function; [0.001, 0.01, 0.1, 1, 10, 100, 1000] values for the BoxConstraint (C) on a logarithmic scale; and [‘scale’, ‘auto’, 0.001, 0.01, 0.1] values for the kernel coefficient (gamma). This space allowed for exploring both linear and non-linear decision boundaries and optimizing the balance between model complexity and fit to the training data. The final hyperparameter values selected were [kernel = ‘gaussian’, C = 1, gamma = 1] [43,44].

## 3. Results

This study aimed to determine smoking addiction levels by analyzing physiological parameters recorded over a two-minute period from 123 volunteers. The analysis employed popular machine learning classifiers (Decision Tree, KNN and SVM) alongside techniques such as k-fold cross-validation and PCA. To address the significant class imbalance in the dataset, both the SMOTE and class-weighting were applied. The optimal results were achieved using 5-fold cross-validation, with the best performance observed when the KNN parameter was set to three neighbors in conjunction with the SMOTE. PCA indicated that the seven original physiological parameters could be reduced to five principal components while retaining 99% of the data variance. For each participant, 60 data points were recorded per parameter, resulting in a total dataset of 7380 instances (123 participants × 60 data points). The classifier performances obtained with 5-fold cross-validation are presented in Table 4.

When the original dataset was used, all models demonstrated high classification performance, with mean accuracy rates above 97%. In this scenario, the Decision Tree model achieved the highest accuracy rate (97.89% ± 0.45). The KNN model similarly exhibited stable performance with an accuracy of 97.75% ± 0.33. Although slightly lower, the SVM model also produced reliable results overall (97.71% ± 0.22). These findings indicate that both distance-based and boundary-based methods can effectively capture the discriminative patterns in the original data distribution.

A significant performance increase was observed across all models following the application of the SMOTE. This demonstrates that mitigating class imbalance directly enhances classification success. The highest performance was achieved by the KNN model, which reached an accuracy of 98.75% ± 0.22, a recall (sensitivity) of 98.74% ± 0.22, and an F1-score of 98.74% ± 0.22. This result confirms that KNN is a highly stable and reliable model under balanced data conditions. Similarly, SVM attained a high accuracy rate (98.62% ± 0.16). The low standard deviation values across all metrics reveal that these models maintained stable performance throughout the 5-fold cross-validation.

In contrast, the results obtained with the class-weighting technique showed partial improvement over the original dataset but were not as effective as SMOTE. The KNN model again achieved the highest accuracy under these conditions (98.52% ± 0.41). However, the Decision Tree model showed a notable decline in accuracy (96.06% ± 1.27), which may be attributed to its higher sensitivity to weight adjustments. The SVM model demonstrated balanced performance with an accuracy of 97.78% ± 0.44. These findings suggest that while class-weighting partially alleviates the imbalance problem, synthetic sampling methods like SMOTE more effectively enhance model generalization.

In overall assessment, data balancing methods significantly improved classification performance. Specifically, the SMOTE + KNN combination provided the highest and most consistent results across all metrics. These results align with studies in the literature, which report that SMOTE-based methods achieve higher success than weighting methods on small to moderately imbalanced datasets [28,45,46].

**Figure 8:**
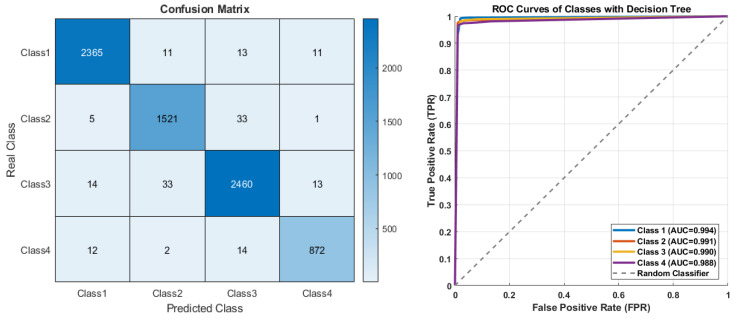
The confusion matrix and ROC curves of Decision Tree on original data.

Figure 8 displays the classification performance of the Decision Tree model on the original dataset, presented through a confusion matrix and ROC curves. Analysis of the confusion matrix reveals that the correct classification rates for all classes are notably high, with misclassifications remaining at a low level. A distinct accuracy advantage is evident, particularly for Class 1, Class 3, and Class 4.

The ROC curves support this finding, showing that the AUC values for all classes exceed 0.98 (Class 1: 0.994, Class 2: 0.991, Class 3: 0.990, Class 4: 0.988). These results demonstrate that the Decision Tree model possesses high discriminatory power on the original data and exhibits strong non-linear separation performance between the classes.

**Figure 9:**
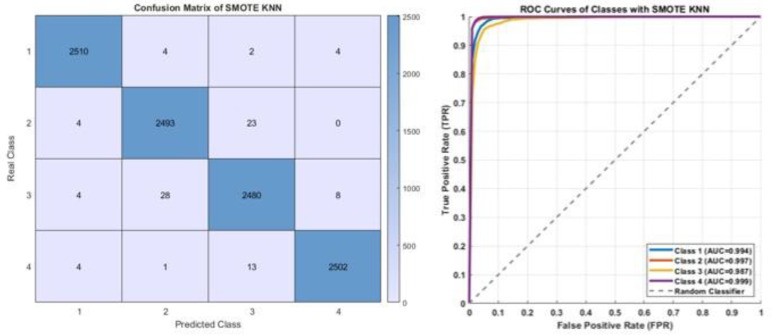
The confusion matrix and ROC curves of KNN on SMOTE.

Figure 9 presents the performance of the KNN model trained on the data balanced with the SMOTE. The confusion matrix reveals that the correct classification rates for all classes are exceptionally high, with misclassifications kept to a minimum. The pronounced dominance of the diagonal values across all classes indicates the model’s successful discrimination between categories. The ROC curves corroborate this finding, showing AUC values above 0.99 for all classes (Class 1: 0.994, Class 2: 0.997, Class 3: 0.987, Class 4: 0.999). These results demonstrate that the KNN model combined with SMOTE exhibits high sensitivity and strong generalization capability, confirming it as a highly effective approach for enhancing performance on imbalanced datasets.

**Figure 10:**
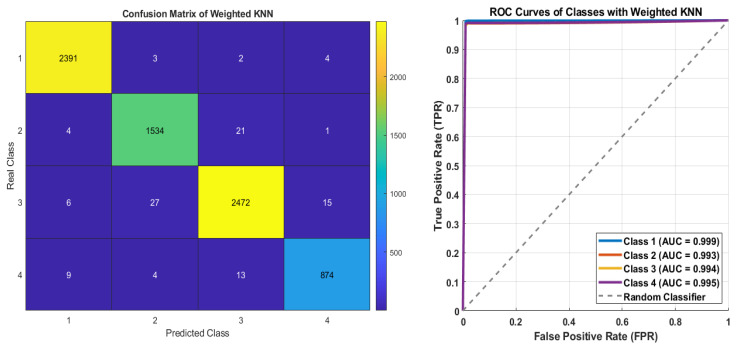
The confusion matrix and ROC Curves of KNN on class-weighting technique.

Figure 10 presents the performance of the KNN model trained with the application of the class-weighting technique. The confusion matrix indicates that high correct classification rates were achieved for all classes, with the number of misclassifications remaining at a very low level. The distinct prominence of the diagonal elements demonstrates the model’s success in discriminating each class individually. Analysis of the ROC curves shows that the AUC values for all classes are above 0.99 (Class 1: 0.999, Class 2: 0.993, Class 3: 0.994, Class 4: 0.995). This result indicates that the weighted KNN model exhibits high discriminatory power by significantly mitigating biases arising from class imbalance. However, minor differences compared to the results obtained with the SMOTE method suggest that the synthetic sampling approach remains more effective in terms of overall accuracy.

Table 5 presents the performance metrics of three different models (Decision Tree, KNN, SVM) following the application of PCA dimensionality reduction, which preserved 99% of the variance. The results demonstrate that applying PCA largely maintained and, in some cases, even enhanced classification accuracy.

According to Table 5, on the PCA-reduced version of the original data, the SVM model achieved the highest accuracy rate (97.30% ± 0.20), followed by the KNN model (96.71% ± 0.39). The Decision Tree model showed lower performance in terms of accuracy (90.08% ± 0.72). This indicates that models sensitive to non-linear boundaries can generalize better within the feature space summarized by PCA.

When the SMOTE method was applied, a significant performance increase was observed across all models. The highest accuracy rate was achieved by the SVM model (98.58% ± 0.23), while the KNN model also demonstrated similarly high performance (98.22% ± 0.28). The Decision Tree model showed only limited improvement even after SMOTE application (92.81% ± 0.52). This result suggests that the reduced sample diversity after PCA may constrain the capacity of tree-based models to define decision boundaries.

When the class-weighting technique was employed, the KNN model again achieved the highest accuracy rate (97.93% ± 0.51). The SVM model similarly exhibited high performance (96.96% ± 0.83), while a substantial decline was observed in the Decision Tree model (86.07% ± 1.64). This decline suggests that the weighting method may cause overfitting or boundary ambiguity, particularly in decision trees.

Overall, on PCA-applied data, the KNN and SVM models stood out with high accuracy, specificity, and F-score values. The SMOTE + PCA combination proved to be the most effective approach for enhancing model generalization on imbalanced datasets. These results align with findings reported in the literature; feature reduction via PCA reduces noise while preserving meaningful variance and, when combined with balanced data, increases the learning stability of models [47].

**Figure 11:**
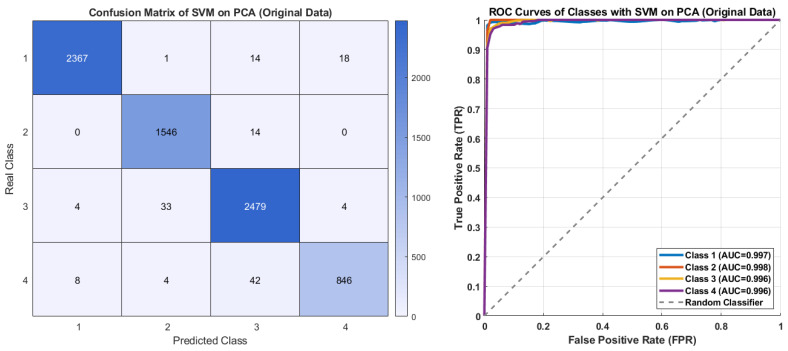
The confusion matrix and ROC curves of SVM on PCA with original data.

Figure 11 displays the classification performance of the SVM model on the PCA-reduced original dataset. It correctly predicted 2367 samples for Class 1, 1546 for Class 2, 2479 for Class 3, and 846 for Class 4. The correctly predicted classification rates for all classes are notably high, with misclassifications remaining at a low level. The ROC curves show AUC values between 0.996 and 0.998 for all classes. These results demonstrate that SVM effectively captured non-linear relationships and maintained robust generalization after PCA reduction.

**Figure 12:**
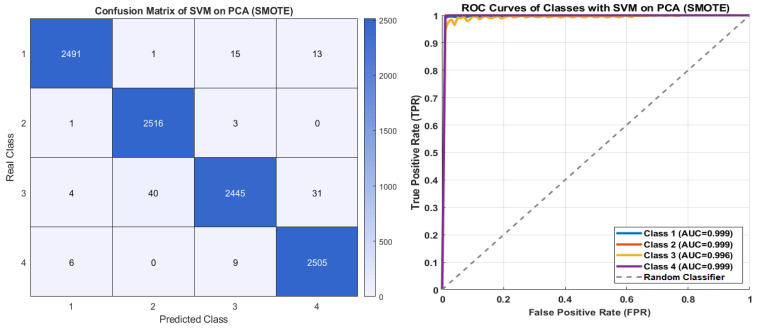
The confusion matrix and ROC curves of SVM on PCA with SMOTE.

Figure 12 presents the confusion matrix and ROC curves of the SVM model on SMOTE. An examination of the confusion matrix indicates that the model achieved very high correct classification rates. Specifically, Class 2 achieved an almost perfect performance with 2516 correctly predicted samples, while Class 1 and Class 4 also demonstrated strong performance with 2491 and 2505 correct predictions, respectively. Even for Class 3, which appeared to be the most challenging, the model correctly classified 2445 samples, indicating consistently high accuracy. The ROC curves show that the AUC values for all classes exceed 0.96 (Class 1: 0.999, Class 2: 0.999, Class 3: 0.996, Class 4: 0.999). These results demonstrate that SVM is a highly effective approach for enhancing performance on imbalanced datasets.

**Figure 13:**
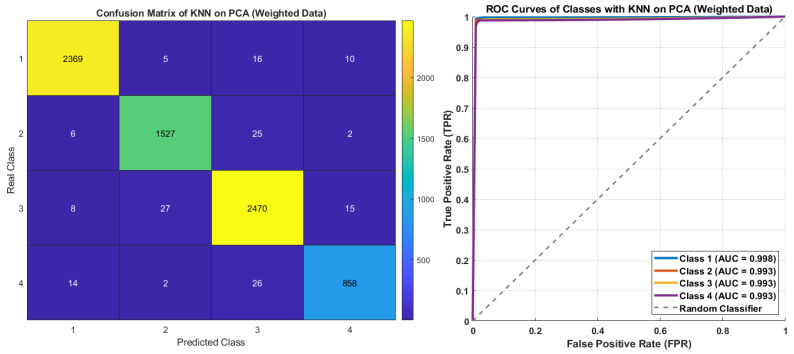
The confusion matrix and ROC curves of KNN on PCA with class-weighting.

Figure 13 presents the confusion matrix and ROC curves of the KNN with PCA and class-weighting. The highest absolute performance was achieved for Class 3, with 2470 correctly classified samples, while Class 4, despite having only 858 samples, achieved 858 correct predictions, indicating exceptionally high precision. Class 1 (2369 correct) and Class 2 (1527 correct) also maintained consistently strong performance. The ROC curves and corresponding AUC values further confirm the balanced performance of the KNN model with class-weighting. An AUC of 0.998 for Class 1 indicates near-perfect separability, while Classes 2, 3, and 4 all achieved AUC values around 0.993, reflecting the model’s consistent sensitivity and fairness across all classes. The KNN model achieved balanced and reliable classification across the four classes, primarily due to the effectiveness of the weighting strategy.

## 4. Discussion

**Figure 14:**
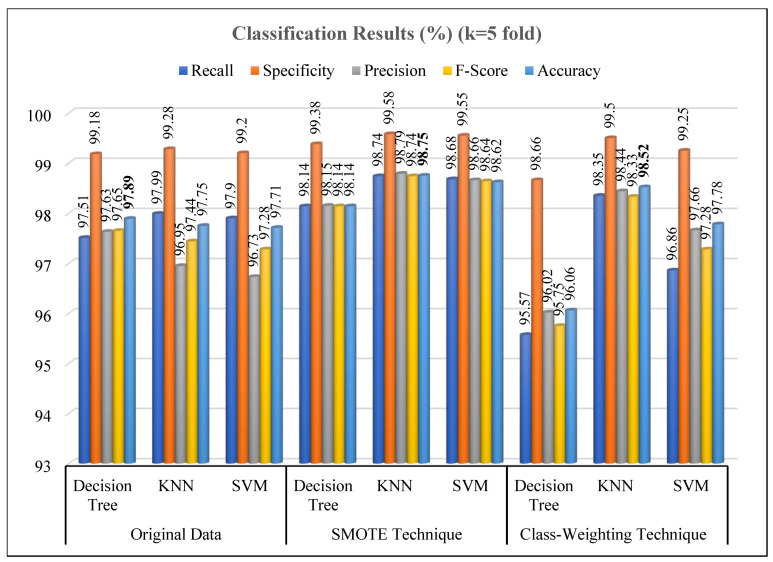
Performance results of classification methods on original data.

**Figure 15:**
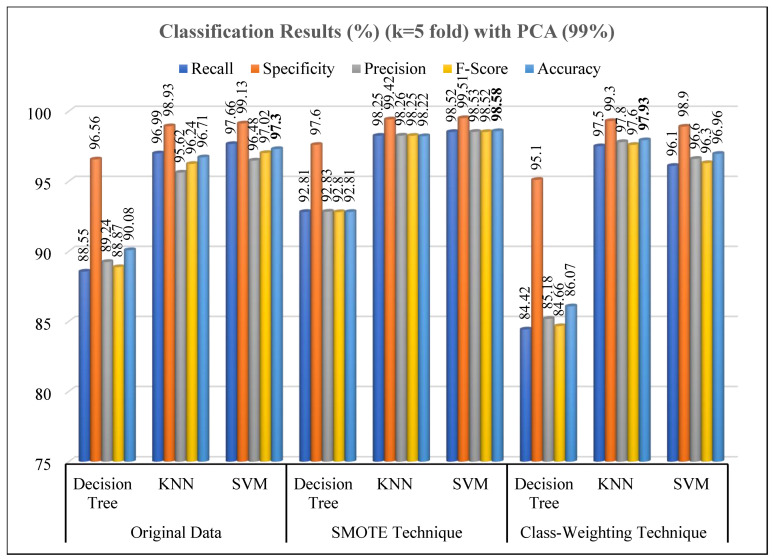
Performance results of classification methods on PCA reduced data.

Figure 14 and Figure 15 present the classification results obtained from the Decision Tree, KNN, and SVM evaluated using the original dataset and under two imbalance-handling strategies, SMOTE and class-weighting approaches. The experiments were conducted both before and after dimensionality reduction using PCA at a 99% variance retention level, and all results were validated using 5-fold cross-validation

As seen in Figure 14, when trained on the original data, all models achieved high classification performance, with accuracies above 97%. The Decision Tree model slightly outperformed others (accuracy: 97.89%), followed by KNN (97.75%) and SVM (97.71%). After applying the SMOTE, a significant improvement was observed in all evaluation metrics, particularly for the KNN model, which achieved an accuracy of 98.75% and an F-score of 98.74%. The enhancement in recall and precision values suggests that SMOTE effectively mitigated class imbalance, providing more robust and generalized learning. This trend aligns with previous studies demonstrating SMOTE’s efficacy in improving minority class detection [28,29,48]. Conversely, the class-weighting technique yielded more variable outcomes. While the KNN model remained strong (accuracy: 98.52%), the Decision Tree experienced a notable decline (accuracy: 96.06%), possibly due to its sensitivity to uneven class penalties. The SVM, on the other hand, maintained stable accuracy (97.78%), consistent with findings indicating its robustness to weighted penalty adjustments [49].

As seen in Figure 15, following PCA-based dimensionality reduction (99% variance retention), the classification performance exhibited minor changes depending on the model type and balancing strategy. Using the original PCA-reduced dataset, the SVM model achieved the highest accuracy (97.3%), followed by KNN (96.71%) and Decision Tree (90.08%). The reduced performance of the Decision Tree indicates a potential sensitivity to feature compression and loss of non-linear feature dependencies. Upon applying SMOTE after PCA, the models achieved their best overall results, especially the KNN classifier, which reached an accuracy of 98.22% and a specificity of 99.42%. The SVM model also exhibited strong generalization (accuracy: 98.58%), confirming that combining SMOTE with PCA enhances class separability by generating synthetic samples in the transformed feature space [29,50]. The class-weighting technique, in contrast, showed more inconsistent behavior. Although KNN maintained competitive accuracy (97.93%), the Decision Tree suffered a substantial decrease (86.07%), highlighting its sensitivity to feature compression and weighting imbalance. SVM, however, remained relatively stable (96.96%), again demonstrating its resilience to class imbalance effects [49].

Overall, both PCA and SMOTE contributed positively to model performance; however, SMOTE consistently outperformed algorithm-level weighting across all evaluation scenarios. The KNN classifier consistently achieved the best trade-off between recall, precision, and accuracy, both with and without PCA.

**Figure 16:**
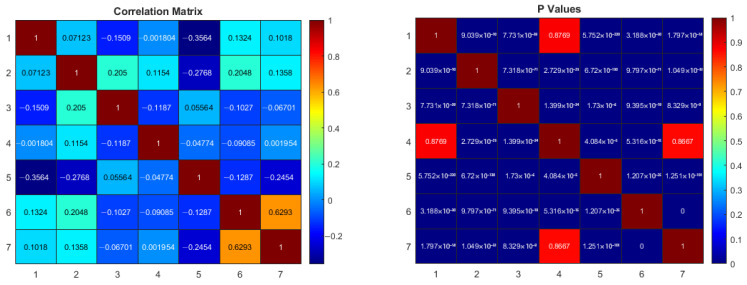
Correlation matrix and *p*-values of original dataset.

The correlation matrix and *p*-values between the parameters in the original dataset are shown in Figure 16. In the correlation matrix, a high positive correlation between the 6th (systolic) and 7th (diastolic) variables can be observed. This is normal because both variables reflect the blood pressure changes in the participants. Additionally, there is no relationship between 1–4 (SpO_2_-PVi) and 4–7 (PVi-diastolic). However, it can be said that there is a low positive or negative correlation between nearly all other variables. When the *p*-values are examined, it is noted that the *p*-values for the 1–4 and 4–7 variables are much greater than 0.05. This means that the correlation between these variables is not statistically significant. For all other variables, the obtained *p*-values are less than 0.05, indicating that the correlations between other variables are statistically significant. However, the values in the correlation table being relatively low in both the negative and positive directions imply that there is a weak, albeit present, relationship between the variables. Furthermore, when determining the main components of PCA, the relationships between variables are utilized. If there is no correlation or very low correlation between the variables, the dimensionality reduction effect of PCA may decrease [51,52]. This is clearly seen in Figure 15. The results in the PCA space provided lower performance for all machine learning algorithms. However, variables with low correlation provide different and independent information for classifiers. This helps the model to learn more general patterns, reduces the risk of overfitting, and makes the model more balanced [51,52,53].

When the recent studies in the literature, as summarized in Table 6, are examined, it is observed that significant research is being conducted in the field of digital health and addiction detection. In these studies, it is generally observed that smoking addiction is addressed at a binary classification level (smoker/non-smoker) using questionnaire data or physiological signals [54,55,56,57,58].

The main originality that distinguishes our study from these is its use of a four-level clinical addiction scale, verified by the clinically accepted Fagerström Test for Nicotine Dependence (FTND). In other words, in this study, subjective questionnaire results are verified by classifying them with high accuracy through objective physiological measurements. This approach demonstrates that the subjective addiction tests used in clinics can be made reliable, objective, and automated with the help of a portable and non-invasive PPG-based system. The main goal of our study is not to examine the acute cardiovascular effects of smoking but rather to present a proof-of-concept for objectively determining individuals’ levels of nicotine dependence with the help of machine learning. This aims to contribute to clinicians managing treatment processes more quickly and effectively.

The portable Massimo Rad-G pulse oximeter device used in our system has ideal features for such an application; besides being portable, it can directly provide the necessary signal dynamics effectively and quickly, rather than providing the raw time series signal.

The most promising practical implication of this study as a proof-of-concept is the high performance and relatively simple structure of the KNN algorithm. Such a model could be integrated into future clinical support systems. For example, a smartwatch or wearable monitoring device could passively collect pulse and respiration data from individuals during their daily lives. It could provide instant feedback that could be used to screen individuals at high risk of addiction or monitor the progress of existing addicts. Clinicians could use the reports from this system, along with traditional questionnaires (FTND), to conduct a more objective and continuous assessment of addiction severity and develop personalized intervention strategies (e.g., adjusting nicotine replacement therapy dosage or the frequency of behavioral therapy sessions). However, for such an application to be realized, the model first needs to be validated in larger and more diverse populations and integrated with a user-friendly interface.

## 5. Conclusions

This study focuses on the fine-grained, multi-level classification of nicotine dependence severity (via FTND scores) using non-invasive, wearable-grade physiological signals from a Massimo Rad-G pulse oximeter. The core novelty of the research lies in leveraging physiological signals to objectively classify addiction levels, thereby addressing the inherent subjectivity of the Fagerström Test for Nicotine Dependence (FTND).

In contrast to the subjective and traditional methods typically used to determine smoking addiction, this study developed an effective and innovative approach to predict addiction levels using physiological parameters (vital signs) and machine learning techniques. The participants’ recorded physiological data were classified by comparing them with their self-reported FTND results, which are based on behavioral and habitual conditions. It is important to note, however, that the FTND itself is a subjective, self-reported tool and does not represent a biochemical gold standard.

To enhance the robustness of the model, the k-fold cross-validation method was applied, and Principal Component Analysis (PCA) was utilized to reduce data redundancy. Furthermore, techniques like SMOTE and class-weighting were implemented to address class imbalance in the dataset. A comparison of performance metrics demonstrated that the KNN classifier consistently achieved the best trade-off between recall, precision, and accuracy, both with and without PCA.

Ultimately, this study provides a proof-of-concept for the feasibility of using machine learning and non-invasive physiological signals to assess nicotine addiction levels. It is believed that this work will offer a valuable contribution to future research in the field of digital health and behavioral diagnostics. However, this investigation should be regarded as a proof-of-concept rather than a clinically validated diagnostic system. Due to the subjective nature of the FTND and the limited dataset, future work should include biochemical validation using established markers such as exhaled CO or cotinine to further strengthen the findings.

## Figures and Tables

**Table 1:** Basic vital parameters.

Class	SpO_2_ Mean	SpO_2_ SD	PR Mean	PR SDNN	RRp Mean	RRp CV%
Class 1	98.1 ± 1.3	1.3	79.8 ± 8.2	36.4	16.8 ± 2.9	16.8
Class 2	95.2 ± 2.9	2.9	92.4 ± 10.1	30.7	20.2 ± 4.1	20.1
Class 3	91.8 ± 4.7	4.7	104.9 ± 12.3	25.2	24.3 ± 6.2	25.3
Class 4	87.1 ± 6.4	6.4	118.3 ± 14.8	20.1	27.9 ± 8.7	31.1

**Table 2:** Heart rate variability analysis.

Class	SDNN (ms)	RMSSD (ms)	LF/HF Ratio	pNN50 (%)	HRV Index
Class 1	36.4 ± 8.2	31.5 ± 7.1	1.52 ± 0.4	25.8 ± 6.3	13.4 ± 2.8
Class 2	30.7 ± 7.4	24.3 ± 6.2	2.01 ± 0.5	17.2 ± 5.1	10.7 ± 2.4
Class 3	25.2 ± 6.8	18.7 ± 5.4	2.68 ± 0.6	11.4 ± 4.2	8.3 ± 2.1
Class 4	20.1 ± 6.1	14.2 ± 4.9	3.41 ± 0.8	6.3 ± 3.1	6.2 ± 1.9

**Table 3:** Respiratory dynamics.

Class	Respiration Rate (Breaths/Min)	RR Coefficient of Variation (%)	Breath Interval SD (Seconds)	Rapid Shallow Breathing Index
Class 1	16.8 ± 2.9	16.8 ± 4.2	1.9 ± 0.4	37.3 ± 8.1
Class 2	20.2 ± 4.1	20.1 ± 5.7	2.9 ± 0.7	48.1 ± 10.4
Class 3	24.3 ± 6.2	25.3 ± 7.8	4.3 ± 1.1	63.9 ± 14.2
Class 4	27.9 ± 8.7	31.1 ± 9.5	5.8 ± 1.6	85.8 ± 19.7

**Table 4:** Performance metrics of methods via 5-fold.

Methods		Performance Metrics (%), (k = 5 Fold)				
		Recall	Specificity	Precision	F-Score	Accuracy
Original Data	Decision Tree	97.51 ± 0.55	99.18 ± 0.16	97.63 ± 0.53	97.65 ± 0.52	**97.89 ± 0.45**
	KNN	97.99 ± 0.33	99.28 ± 0.11	96.95 ± 0.48	97.44 ± 0.39	97.75 ± 0.33
	SVM	97.90 ± 0.26	99.28 ± 0.08	96.73 ± 0.32	97.28 ± 0.26	97.71 ± 0.22
SMOTE Technique	Decision Tree	98.14 ± 0.31	99.38 ± 0.10	98.15 ± 0.31	98.14 ± 0.31	98.14 ± 0.31
	KNN	98.74 ± 0.22	99.58 ± 0.07	98.79 ± 0.24	98.74 ± 0.22	**98.75 ± 0.22**
	SVM	98.68 ± 0.12	99.55 ± 0.05	98.66 ± 0.16	98.64 ± 0.15	98.62 ± 0.16
Class-Weighting Technique	Decision Tree	95.57 ± 1.52	98.66 ± 0.41	96.02 ± 1.44	95.75 ± 1.41	96.06 ± 1.27
	KNN	98.35 ± 0.63	99.5 ± 0.11	98.44 ± 0.58	98.33 ± 0.51	**98.52 ± 0.41**
	SVM	96.86 ± 0.73	99.25 ± 0.26	97.66 ± 0.40	97.28 ± 0.59	97.78 ± 0.44

**Table 5:** Performance metrics of methods with PCA via 5-fold.

Methods		Classification Results (%) with PCA (99%), (k = 5 Fold)				
		Recall	Specificity	Precision	F-Score	Accuracy
Original Data (Reduced by PCA)	Decision Tree	88.55 ± 0.86	96.56 ± 0.25	89.24 ± 0.87	88.87 ± 0.85	90.08 ± 0.72
	KNN	96.99 ± 0.40	98.93 ± 0.13	95.62 ± 0.55	96.24 ± 0.46	96.71 ± 0.39
	SVM	97.66 ± 0.16	99.13 ± 0.06	96.48 ± 0.38	97.02 ± 0.27	**97.30 ± 0.20**
SMOTE Technique	Decision Tree	92.81 ± 0.53	97.60 ± 0.17	92.83 ± 0.53	92.80 ± 0.53	92.81 ± 0.52
	KNN	98.25 ± 0.26	99.42 ± 0.09	98.26 ± 0.26	98.25 ± 0.26	98.22 ± 0.28
	SVM	98.52 ± 0.25	99.51 ± 0.08	98.53 ± 0.25	98.52 ± 0.22	**98.58 ± 0.23**
Class-Weighting Technique	Decision Tree	84.42 ± 2.73	95.10 ± 0.61	85.18 ± 1.85	84.66 ± 2.19	86.07 ± 1.64
	KNN	97.5 ± 0.16	99.3 ± 0.20	97.8 ± 0.41	97.6 ± 0.75	**97.93 ± 0.51**
	SVM	96.1 ± 1.3	98.9 ± 0.31	96.6 ± 0.92	96.3 ± 1.11	96.96 ± 0.83

**Table 6:** Recent studies in the literature.

Study	Data Source	Machine Learnings	Classes	Accuracy	Paticipant	Focus
Kharabsheh et al. (2019) [54]	Survey Data (WTSQ)	Dec.Tree, SVM, k-Star, Naive Bayes	3	82%	108	Survey-based dependence classification
Choi et al. (2021) [55]	Survey Data (NYTS)	RF, LASSO	4	73.42%	6511	Risk factor analysis in adolescents
Issabakhsh et al. (2023) [56]	Basic Vital Signs	Random Forest, XGBoos	2	81%	5000	Biomarker-based smoking status classification
Aishwarya et al. (2025) [57]	Clinical/Biological Data	RF, LR, Dec.Tree, KNN, CatBoost, ANN	2	85%	2000	Binary smoking status classification (Smoker/Non-smoker)
Qananwah et al. (2025) [58]	PPG+EKG	Gaussian Process Regression	3	99.7%	84	Predicting smoking’s acute effect on blood pressure
This Study (2025)	PPG (SpO_2_, PR, RRp, Pi, Pvi, BP)	Dec.Tree, KNN, SVM	4	98.75%	123	PPG-based multi-level nicotine dependence severity assessment

## Data Availability

The authors confirm that the data to support the findings of this study are available upon request to the corresponding author.
